# Mental workload task modeled on office work: Focusing on the flow state for well-being

**DOI:** 10.1371/journal.pone.0290100

**Published:** 2023-09-06

**Authors:** Chie Kurosaka, Hiroyuki Kuraoka, Takashi Maruyama

**Affiliations:** 1 Department of Human, Information and Life Sciences, School of Health Sciences, University of Occupational and Environmental Health, Kitakyushu, Japan; 2 Department of Occupational Hygiene, School of Health Sciences, University of Occupational and Environmental Health, Kitakyushu, Japan; 3 Department of Physiology, School of Medicine, University of Occupational and Environmental Health, Kitakyushu, Japan; University of Cadiz: Universidad de Cadiz, SPAIN

## Abstract

This research aimed to objectively evaluate the optimal state of desk work (flow state) through physiological measurements and use the data to support workers’ mental health and well-being. We suppose that the flow state evaluation in real-time can contribute to a concentrated work environment, improved work efficiency, and stabilize worker’s minds. This study reports on the development of the mental task modeled on daily work for the physiological measurement experiment. In the first phase of the research, a field survey was conducted with 55 desk workers to understand the details of their jobs and develop suitable mental tasks. Further, the relationship between daily work content and subjective stress was clarified. In the second phase, based on the results of the field survey, a task inducing the flow state was developed for practical use. Through empirical experiments with 35 participants (22 adults and 13 students), the developed task was evaluated for its usefulness and possible issues by examining the relationships among subjective assessment, task performance, degree of flow state, and individual characteristics. The study results showed that the proposed mental task developed in this study constitutes practical work that can be used for concentrated and goal-directed efforts. The task also demonstrated the property of inducing a flow state. Further, the results suggest that it is necessary to adjust the task difficulty level and implement effective feedback methods to induce the flow state more effectively.

## Introduction

Well-being at work contributes not only to maintaining workers’ health but also to increasing their productivity. “Good Health and Well-Being” is one of the Sustainable Development Goals adopted by the United Nations in 2015; parallelly, the movement to enhance workers’ health and work-life balance has become a global trend. A program launched by the Japanese Cabinet Office, Moonshot for Human Well-Being, also includes the enhancement of mental comfort and vitality [1]. Positioning these perspectives in present-day life becomes important to be able to actualize the respective goals. Today’s society is referred to as a “Ubiquitous Computing Environment,” meaning that diverse information and communications technology (ICT) devices are part of our daily lives. Internet of Things technology and wearable devices are being widely used as familiar equipment [2], and abundant, varied data are collected in daily life through environmental indexes, behavioral assessments, and physiological parameters. In this context, examinations to visualize health conditions are also in progress, with some examples including physical activity data measured by devices used on the lumbar region or wrists [3] and evaluations of physical activity using big data [4]. Strain et al. [5] showed the possibility of creating a framework for personalized health care by linking physical activity measured through wearable devices to energy expenditure. ICT devices appear to be making a breakthrough in their applicability, from mere office work supplies or communication tools to those capable of supporting human safety and health. Hence, a valuable goal to pursue would be to discover how ICT devices can provide optimal functionality.

Various studies have been conducted on the estimation of human mental states using physiological responses. Although there is growing interest and demand for the estimation of routine mental states using physiological variables measured by wearable devices, several problems emerge with respect to applying the findings of laboratory experiments to the practical world; for example, the failure to measure and detect physiological changes that are not related to mental states, such as with physical activity and conversation. These issues are expected to be addressed in the future with the development of devices and ICT technology. A major concern is whether the same physiological responses occur both in the laboratory and real-world contexts. We believe that the root of the question resides in the mental workload used in the experiments. The International Organization for Standardization (ISO 6385) regards work stress as an external load, defining it as the “sum of those external conditions and demands in the work system which act to disturb a person’s physiological and/or psychological state.” Moreover, four workload elements are considered to constitute a good predictive measure: analytic, physiological, subjective, and behavioral factors [6]. Based on the above definition, previous studies have attempted to estimate mental stress, mostly negative stress, by combining physiological responses and subjective assessments during a mental workload under controlled conditions and constrained behavior. Since physiological responses are influenced by factors such as age and gender, it is necessary to collect data from a diverse range of participants. However, it is difficult to conduct simultaneous physiological measurement experiments on multiple subjects during the mental workload, and these experiments are often conducted with a restricted range of participants’ characteristics. Additionally, the verification process must involve repeating the experiment while changing participant characteristics and various conditions. For these reasons, laboratory experiments often use simple mental tasks that can be set up with various conditions and measurements of performance, such as the Stroop task, mental arithmetic, and puzzles [7–9] to ensure the reproducibility of the same experiment. Such mental tasks have been developed by focusing on attentional resources such as specific cognitive tasks (e.g., perception, memory, reasoning) and perceptual-motor tasks (e.g., tracking, mirroring description). However, it is unclear whether the same physiological responses observed in the fundamental laboratory experiments will occur in the complex everyday life since these tasks are far from daily work. Therefore, it is necessary to develop mental tasks that mimic daily work and validate past findings using the task to discuss the relationship between mental states and physiological responses in daily life.

In addition, our interests extend to positive mood states for well-being, as we believe that it is better to focus more on these positive states than the negative aspects when evaluating human mental states. Work engagement is one of the most important factors related to worker well-being, defined as a consistent state of mind that persists for a period of time. Work engagement is not a temporary state directed toward a specific object or event, but a sustained, holistic feeling directed toward work, and is complexly related to a multitude of factors. Therefore, when considering strategies to enhance individual work engagement, it is crucial to delve into the specifics. According to Schaufeli et al. [10] and Kahn [11], work engagement comprises three components: Vigor = physical-energetic, Dedication = emotional-energetic, and Absorption = cognitive-energetic. In addition, Schaufeli et al. [10] defined Absorption as a state of being fully concentrated and happily engrossed, whereby time passes quickly, and one has difficulties with detaching herself/himself from the work she/he is engaged in. Therefore, we have focused on "flow state," which is a short-term mental state characterized by deep concentration and complete immersion in a sense of enjoyment of an activity process. Although a direct causal relationship between work engagement and flow state is not always clear, they are considered mutually interrelated as they are both characterized by sense of enjoyment, achievement, high concentration, and motivation at work. The flow state is experienced when both the level of challenge and skill are matched to a high degree. Consequently, it can contribute to high productivity and satisfaction [11],The flow state is also referred to as “the zone” in sports, and the flow experience is considered to maximize players’ abilities and cognitive skills [12, 13],In their study, Daniela et al. [14] revealed a positive relationship between work engagement and flow level. Further, they found that workers with high levels of flow at the work are more likely to be engaged, have more sense of enjoyment, and ability to focus on tasks, even in adverse situations, regardless of work environmental factors under the umbrella of autonomy support. Furthermore, Wang et al. [15] proposed a theoretical framework model of employee flow state promotion and high-involvement human resource management (HIHRM) from an organizational management perspective. They emphasize the importance of linking HRM and employees’ flow state to enhance employees’ well-being and performance to an optimum level. Flow at work has been studied through interview, questionnaire, and experience simple method (ESM) [16–18], which require retrospectively reconstructed past experience and aggregate an overall picture of flow experience. These methods require respondents to evaluate the flow at periodic intervals, which may interfere with their work. It is also impossible to know the duration and exact frequency of the flow state. Therefore, we believe that it is valuable to explore methods for estimating the real-time flow state and that physiological measurements and/or work performance obtained from wearable sensors and ICT devices will be useful in achieving it. However, few physiological measurement studies have been conducted for flow estimation, thus it is necessary to search for useful physiological indices in laboratory experiments. Furthermore, developing tasks that can be used in the laboratory is required.

As mentioned above, to apply the findings of laboratory experiments to the real world, it is necessary to conduct verification in tasks that are more similar to practical work, rather than in mental arithmetic problems or puzzles. Although there is a wide variety of tasks in practical work, given the consideration of applying data using ICT devices, our first step is to investigate the types, frequency, and difficulty levels of tasks by focusing on desk workers who are less influenced by physical movements. To induce a flow state, it is important to have a high level of balance between challenge and skill and to receive feedback on the work. For example, tasks such as preparing conference materials and writing papers are considered to be candidates for mental tasks to be developed. This study focused on the following two specific objectives: (1) To identify the frequently performed tasks in daily work of desk workers; (2) To develop a useful mental task that induces a flow state for laboratory experiments.

## Methods

### Field research: Daily work identification

A field survey was conducted with 55 desk workers, including 12 males and 43 females, aged 23–63 years (mean±SD age: 43.6±12.1), to gather information about their job details. Participants selected a convenient day during the survey period and recorded the tasks they performed from the beginning to the end of the workday, particulars of which are presented in Table 1. In addition, the levels of mental stress and work achievement were subjectively evaluated every 30 minutes on a five-point scale (0: none to 4: very high). Participants were asked to complete a face sheet that sought details regarding their gender, age, length of employment, number of working years in the current department, childcare/caregiving experience, sleep duration, and their drinking the night before the survey date. Furthermore, the Work Functioning Impairment Scale (WFun) [19] was administered to investigate the degree of work dysfunction due to health problems. The Wfun is a questionnaire in which participants answer seven work-related questions, for example, “I could not do my job properly,” on a five-point scale (1: not at all to 5: almost every day). The WFun is evaluated using the combined score of its seven items. A higher score means a greater degree of occupational dysfunction. This research was approved by the Institutional Review Board of the University of Occupational and Environmental Health (Approval No. H29-229) and the research was conducted in accordance with the guidelines based on the Declaration of Helsinki. All participants completed written informed consent forms after receiving written descriptions of the survey.

**Table 1:** Type of work.

Classification	Content	Examples
CW	Creating the documents and designing from scratch	Preparing the planning document, programming
IP	Interactive communication	Attendance at meeting
ID	Filling in or input of data into designated forms	Preparing order slips
ED	Typographical error detection, checking the consistency of numbers and amounts	Checking the submitted forms
MW	Sorting, photocopying, sealing, and stapling	Photocopying meeting documents
IG	Gathering information from the internet, catalogs, one-way inquiries, etc.	Route and expense search for business trips
OT	(Please be more specific)	Break, cleaning

*Notes*: CW: Creative work; IP: Interpersonal; ID: Input data; ED: Error detection; MW: Monotonous work; IG: Information gathering; OT: Other.

First, participant characteristics were tabulated. The relationship between the WFun scores and childcare/caregiving experience (presence or absence) and sleep duration was analyzed using a t-test and correlation analysis. Next, the frequency of occurrence of each job description per 30 minutes was summarized, and the trends in subjective stress and work achievement ratings (scored from 1 to 5) were calculated. Subjective stress and work achievement ratings were calculated for each job description, and one-way analysis of variance (ANOVA) and Tukey’s method were used to test for significant differences (SPSS Statistics 19). In addition, χ^2^ test and residual analysis were conducted to examine the relationship between WFun and work content.

### Mental workload proposal based on field research

According to the results of the field survey to identify daily work, jobs that included creation and interpersonal elements scored higher in terms of frequency of occurrence, subjective stress value, and work achievement level. The conditions for inducing flow include the person perceiving a balance between the challenges of a situation and their own skills, with both operating at a high level that is above average for the individual. Additionally, the task should provide unambiguous feedback, and must be continuous and enjoyable. We considered the task of writing e-mails as a mental task that potentially includes all of these features and conditions. Additionally, we examined the structure of the mental task so that it would be experimentally condition-controllable and suitable for the measurement of physiological responses and performance.

First, the e-mail creation situations were developed based on interviews with five working adults who communicated through e-mail in their daily work. The situation condition consisted of six types of e-mail messages, namely, greeting, request, report, consultation, apology, and refusal, based on e-mail messages sent and received as part of the participant’s daily work. To avoid bias, the five desk workers familiar with e-mail were added as evaluators, while all the ten workers assessed the difficulty of each trial-created situation (1: very easy, 2: easy, 3: fair to easy, 4: fair to difficult, 5: difficult, 6: very difficult). After repeated modifications and validation, two sets of six conditions with equal difficulty levels were prepared (S1 Table).

We presented six fictitious situations to the participants to modulate the task’s difficulty level. After the participants understood the situation, they were given 15 minutes to compose an e-mail. In addition, the participants were instructed as follows: 1) Write an e-mail as a fictional character; 2) You can decide the order in which to work on each of the six fictitious situations; 3) The e-mail can be saved as a draft; 4) Completed e-mails are not actually sent (only their status is changed to “completed”).

As indicators of work performance, we recorded the number of (A) e-mails started; (B) e-mails completed; (C) characters entered; and (D) characters entered per e-mail (D = C/A). The task time was set to a limited time, 15 minutes, to make it extremely difficult to complete the e-mails for all conditions, based on the necessity of continuing the mental workload until the end of the experiment. As indicated above, the e-mail was not actually sent; when a participant completed an e-mail and clicked the “Finish Button,” the edit mode was disabled.

The number of completed e-mails and input characters were displayed on the screen to provide participants feedback on task progress. Computers have some text input aids such as input history and predictive text input; however, all such functions were turned off during the experiment. Further, internet searches were also disabled. As an alternative, a series of phrases for each situation could be displayed on the screen. Participants created e-mails as fictitious people in the experiment, and the signatures were preset and uneditable. Participants composed only the subject and body of the e-mail.

We thus implemented a mental workload task in Microsoft Excel 2016 that satisfied all the requirements described above (S1 Fig). Participants could switch between each situation themselves by changing the six Excel sheets.

### Mental workload validation procedure

To examine the usefulness of the proposed mental task, we conducted an e-mail writing exercise involving 35 participants who provided their consent prior to participation. In total, 35 participants consisting of 22 businesspersons (male: female ratio = 6:16, ages: one in 20s, five in 30s, 13 in 40s, and three in 50s) and 13 students (male: female ratio = 6:7, ages: all in 20s) completed the task. The businesspersons were all desk workers who are using e-mail as part of their daily work. Ten of them always used it. In contrast, post-experiment interviews revealed that all students had little or no experience with e-mail because of the popularization of social networking services. Before the experiment, two personality tests (Yatabe-Guilford personality test: YG test; Kwansei Gakuin daily life questionnaire: KG questionnaire) were administered.

Before writing the e-mails, participants read six situations based on which they were required to compose the e-mails (S1 Table: A1 to A6 or B1 to B6) and respond regarding the difficulty level of the task on a six-point scale (1: very easy, 2: easy, 3: fair to easy, 4: fair to difficult, 5: difficult, 6: very difficult). Participants were required to write as many e-mails as possible within 15 min, and they had the flexibility to show and select from six situations at their convenience. After writing the e-mails, we administered the NASA Task Load Index (NASA-TLX) [20] which comprises six subscales: Mental demand (MD), Physical demand (PD), Temporal demand (TD), Own Performance (OP), Effort (EF), and Frustration level (FR). Subsequently, we used the Flow State Scale Japanese version (FSS) [21, 22] and the Duration Judgment Ratio (DJR) [23]. The FSS was rated on a 7-point scale (1: not at all applicable, 2: not applicable, 3: not much applicable, 4: Neutral, 5: a little applicable, 6: applicable, 7: very applicable) for 36 items. Regarding the DJR, participants were asked to rate their perception of the e-mail writing time compared to 15 minutes based on the VAS method (from 0: shortest to 100: longest). Following a break, e-mail writing and subjective assessments were repeated under different conditions, and the task progress level and phrase list for the business mail were displayed in the second session. Task progress was offered as feedback to participants and phrase lists were provided to help smooth the task process, these were ultimately considered to induce a more flow state. Since each participant repeated the task twice, two pairs of six situations (A and B) for composing e-mails were prepared, and the participants were randomly allocated to constitute equal proportions of working adults and students. Of the participants, 19 (including seven students) engaged in Condition A in the first session and Condition B in the second session. The other participants performed the task in reverse order. Participants were informed that they did not have to complete all six e-mails within 15 minutes, they were free to write e-mails in any order, and they could proceed based on their own judgment in case of any ambiguity during the writing process.

This experiment was approved by the Ethics Committee of the University of Occupational and Environmental Health, Japan (Approval No. H30-204), and the experiment was conducted in accordance with the guidelines based on the Declaration of Helsinki. After receiving written descriptions of the experiment, all participants completed written informed consent forms.

### Analysis for mental workload validation

The difficulty of each situation was scored on a six-point scale and the average for all trials was calculated. The difficulty averages were calculated for each progress status, condition, order, and type of occupation. These averages were compared by using repeated measures ANOVA, multiple comparisons (Holm method), and paired t-test (SPSS Statistics 19). The number of unfinished e-mails, the number of completed e-mails, the number of characters entered, and the number of characters per e-mail were calculated as work performance indexes. The number of emails was tested for significance using the Wilcoxon matched pairs signed-rank test for Condition (A/B) and Session (First/Second), the Mann-Whitney U test for participants (Worker/Student), and the paired t-test for the number of characters (SPSS Statistics 19). In the analysis of participants, the mean value of the first and second sessions for each participant was calculated and compared.

NASA-TLX scores for all subscales ranged from 0 to 100 and the adaptive weighted workload (AWWL) [24] is calculated from the individual subscale scores. The task performance and NASA-TLX were also analyzed using paired t-test for each condition, session order, and type of occupation. Regarding the flow scores, 36 items were grouped into nine subscales of four items each (Clear goals, Unambiguous feedback, Self-oriented experience, Concentration, Sense of control, Transformation of time, Autotelic experience, Balanced challenge and skill, and Loss of self-consciousness), and the threshold score for each subscale was set at 16 points because the score of 4 is indicative of neutral status. Furthermore, the flow score is obtained by adding together the nine subscales. Therefore, a flow state was considered to have occurred when the flow score (total score) was higher than 144 points. For each subscale score and flow score, the number of cases exceeding the threshold was calculated. The mean values of the FSS score and work performance indexes between both sessions were calculated, and their relationships were calculated using a correlation matrix with each item of the two personality tests. The DJR scores ranged from 0 to 100, with a score of 50 indicating that the time perceived by the participant on the task is equal to the actual time, with smaller scores indicating that participants perceived the time spent on the task as shorter than the actual time spent on the same. DJR was evaluated by comparing the scores to 50 in each session using a one-sample t-test (SPSS Statistics 19). The relationship between NASA-TLX, work performance, and flow was analyzed by obtaining a correlation matrix.

## Result

### Daily work identification

The working time and Wfun score are shown in Table 2. No bias was found related to the length of employment, number of working years in the current department, and sleep duration (Table 2). The participants worked 8 hours regularly, from 8:30 to 17:15 (including a 45-minute break). Seven of the 55 participants worked for lesser hours (three participants: 8:30 to 16:00; four participants: 9:00 to 16:00, 9:00 to 15:00, 9:30 to 15:30, and 8:00 to 12:00).

**Table 2:** Working time and WFun score on survey day (mean±SD).

	Male	Female	All
Years of employment (yrs.)	17.2±11.4	15.5±13.7	15.9±13.3
Years of working in the current department (yrs.)	3.0±2.6	4.2±4.6	3.9±4.3
Working hours on survey day (hh:mm)	10:00±1:31	9:15±1:49	9:25±1:46
Working hours on survey day excluding break time (hh:mm)	9:00±1:31	8:18±1:45	8:27±1:43
WFun score	16.0±5.0	15.1±6.1	15.3±5.9

*Note*: WFun: Work Functioning Impairment Scale.

The average working hours were 9 hours and 25 minutes (excluding break time: 8 hours and 27 minutes), and three participants worked overtime after 19:00. The WFun score results were 24 (43.6%) for no problem, 21 (38.2%) for mild, 7 (12.7%) for moderate, and 3 (5.5%) for severe; no significant relationship was noted between the scores and sleep duration (r = -0.149) or caregiving/childcare experience.

The frequency of occurrence and number of participants in each job category are shown in Table 3. In the case of multiple types of work being performed simultaneously, each task was counted as a separate operation.

**Table 3:** Number of appearances and number of people for each work task performed on the survey day.

Type of Work	Total number of performed tasks	Ratio (per 1,248 tasks)	Number of people who performed the task	Ratio (per 55 persons)
CW	369	29.6%	42	76.4%
IP	234	18.8%	44	80.0%
ID	227	18.2%	32	58.2%
ED	133	10.7%	34	61.8%
MW	110	8.8%	27	49.1%
IG	53	4.2%	16	29.1%
OT	122	9.8%	54	98.2%

*Notes*: CW: Creation work, IP: Interpersonal, ID: Input data, ED: Error detection, MW: Monotonous work, IG: Information gathering, OT: Other.

**Fig 1:**
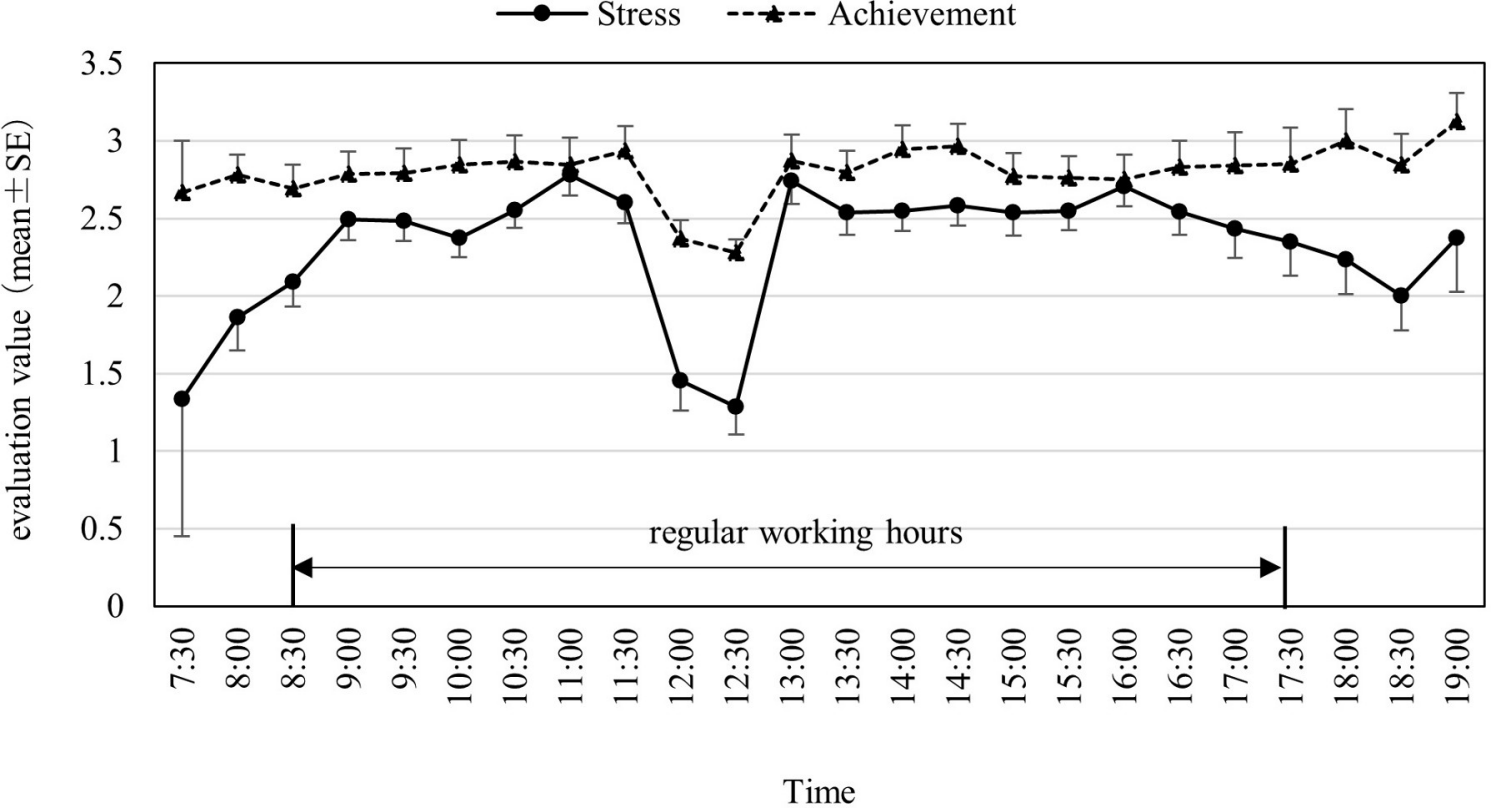
Changes in the average of subjective stress and work achievement levels.

Regarding each job type, the frequency was the highest for creation work (29.6%), followed by interpersonal work (18.8%) and input work (18.2%). Creation work predominantly involved preparing meeting materials and application documents, while interpersonal work was mostly performed during meetings and while interacting with visitors. More than 70% of the workers were engaged in creation and interpersonal work. Fig 1 shows the trends of subjective stress and work achievement ratings over time during work hours. These scores remained almost constant. Subjective stress levels were lower during the lunch break, but there was no difference between the morning and afternoon hours. Ten participants (18.2%) had an average subjective stress rating of more than 3, and two of them had a rating of more than 4. One of the two participants who had particularly high-risk ratings always experienced considerable stress during work, but his/her work achievement level was also high, with a WFun score judged as moderate. The other participant had an average WFun score and felt high stress temporarily during the interpersonal work period.

**Fig 2:**
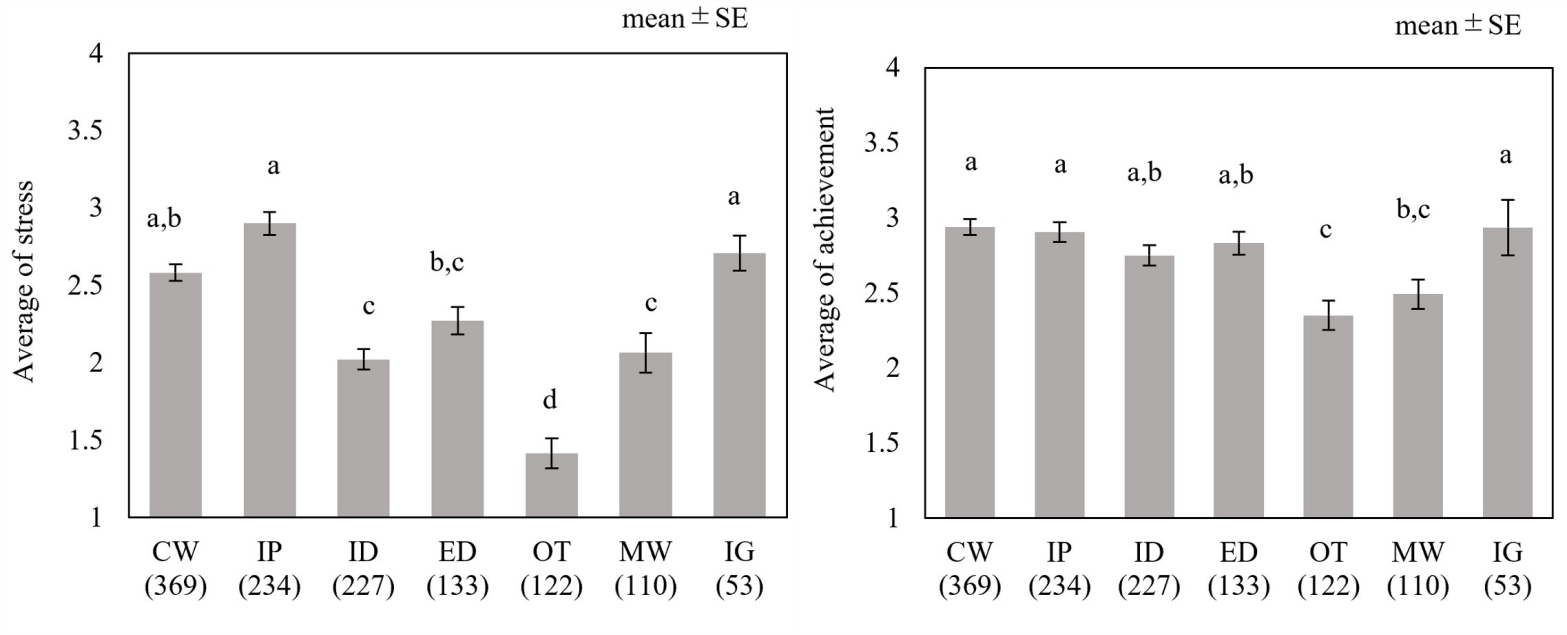
Subjective evaluation of each type of work.

Fig 2 shows the results of the subjective stress score and work achievement level evaluation for each job content. The alphabets indicated homogenous subset results based on Tukey’s honest significant difference (p<0.05).

**Fig 3:**
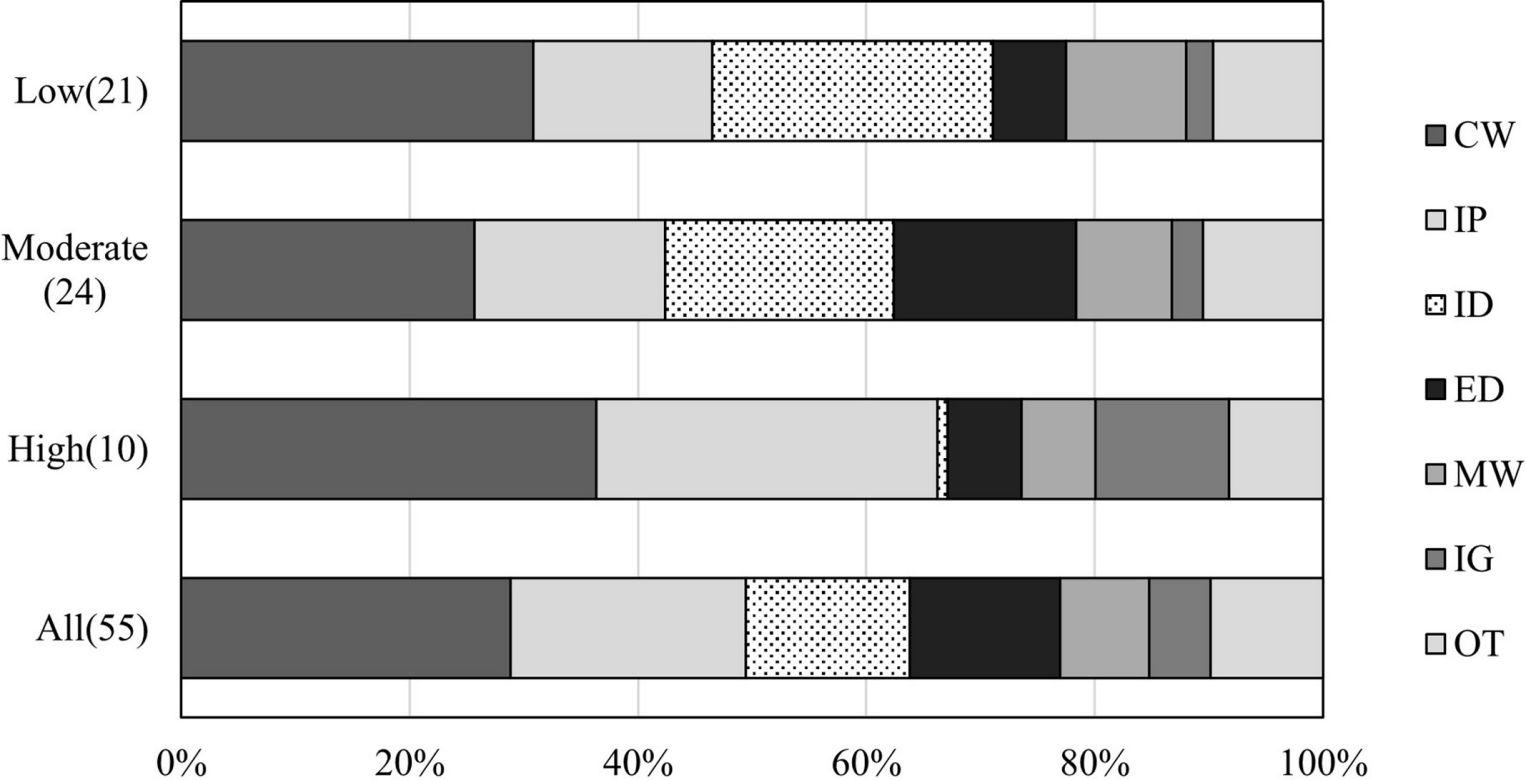
Residual analysis for the relationship between the WFun score and type of work.

Stress evaluation for creation work, information gathering, and interpersonal work yielded significantly higher values than for other types of work. No significant differences were noted in the work achievement rating value for each type of work; however, the value of monotonous work was lower than those of other types. Fig 3 presents the residual analysis results for the relationship between the WFun score and type of work.

The chi-square test results indicated a significant relationship between the WFun score and type of work. The residual analysis showed that the high WFun score group had a higher frequency of creation work, input work, and information gathering. Meanwhile, the moderate WFun score group had a significantly lower frequency of creation work and a higher frequency of error detection.

### Validation of the mental workload

**Fig 4:**
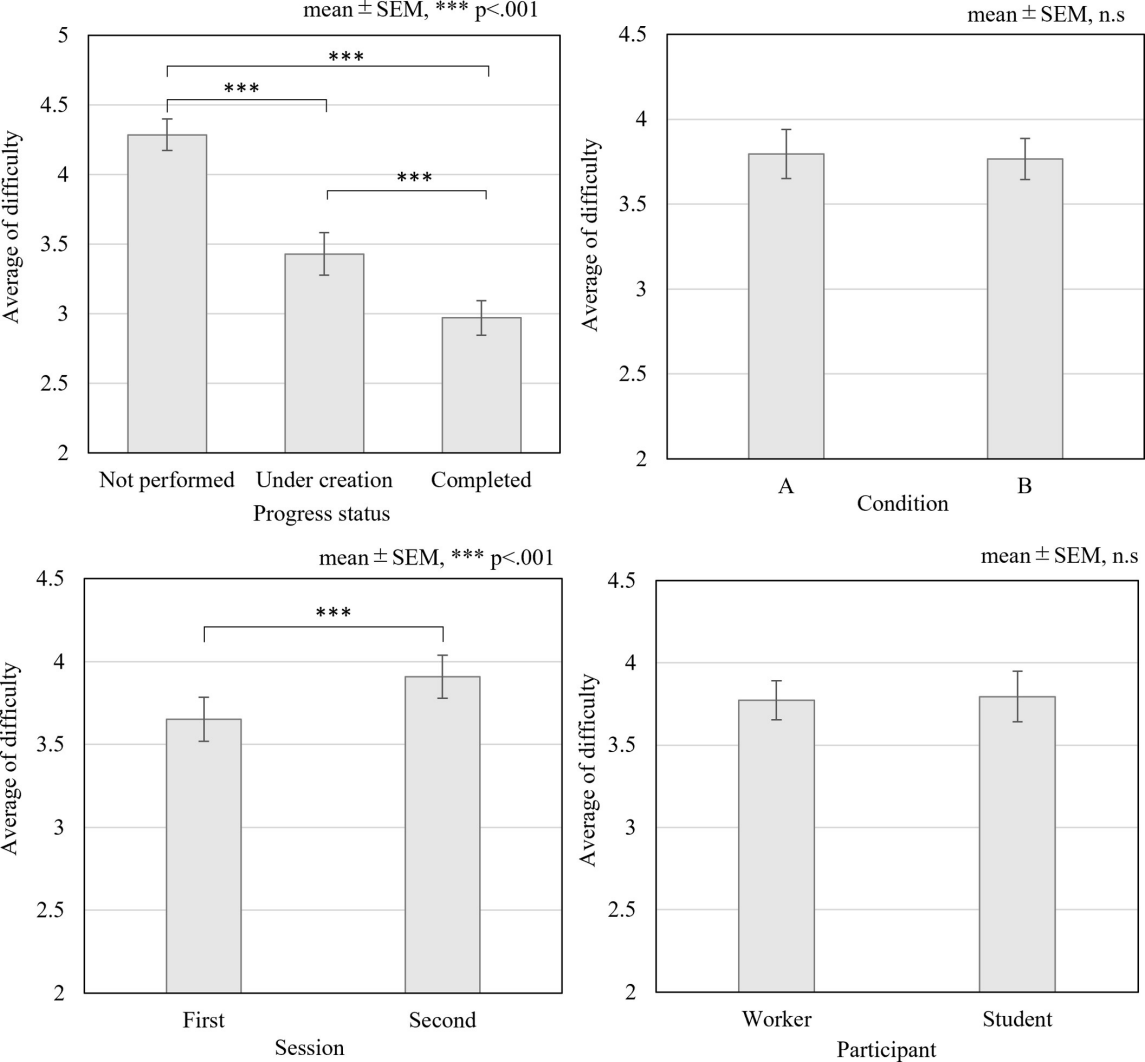
Average of task difficulty.

The mean and standard deviation of task difficulty for all trials was 3.78±1.38. The mean difficulty by group (task progress status, condition, order, and type of occupation) is shown in Fig 4. A significant difference was noted for each task progress status (F = 49.86, df = 2, η^2^ = 0.475, p<0.001) , and the Holm method results revealed that participants had worked on and completed the tasks with lower difficulty. No significant differences were found for each condition (p = 0.719) and each type of occupation (p = 0.0910). Meanwhile, a significant difference was observed for each session order (t = -3.938, df = 34, p = 0.0039), and task difficulty in the second session was higher than that in the first.

### Performance and the NASA-TLX

Table 4 shows the average number of e-mails and characters entered for each group. The number of unfinished e-mails refers to the number of e-mails that were in the process of being written and does not include the number of completed e-mails. The number of composed and completed e-mails increased significantly in the second session. The number of completed e-mails was also significantly different between working adults and students.

**Table 4:** Number of e-mails and characters entered.

	All	Condition			Session			Participant		
		A	B	*p*	First	Second	*p*	Workers	Students	*p*
Unfinished e-mails	0.87± 0.44	0.89	0.86	0.739	0.86	0.89	0.739	0.82	0.96	0.452
Completed e-mails	1.8± 0.97	1.69	1.91	0.122	1.66	1.94	0.048*	2.05	1.38	0.027*
Characters entered	394.89±158.06	395.63	394.14	0.916	375.29	414.49	0.003**	405.48	376.96	0.371
Characters per e-mail	152.11±49.83	156.64	147.58	0.331	152.14	152.08	0.994	141.82	169.53	0.074

Notes:

* p<0.05

**p<0.01.

The NASA-TLX scores and the statistical results are shown in Table 5. Mental demand and Effort were higher than 70, and students had significantly higher Mental demand than working adults. The number of trials with a score rating below 50 was very few: 10 trials for Mental demand (14.7%) and 5 trials for Effort (7.4%). Meanwhile, Temporal demand, Effort, and Frustration were not high. Own performance was significantly higher in the second session than the first (p = 0.01), but the absolute value was lower. The work performance index was obtained by assigning two points to completed e-mails and one point to unfinished e-mails, and the correlation coefficient between the NASA-TLX index and the NASA-TLX index was calculated. Among the subscale of NASA-TLX, only Own performance has a negative significant correlation with the task performance index (r = -0.427, p<0.001) and entered characters (r = -0.444, p<0.001).

**Table 5:** Results of the NASA-TLX.

	All Mean±SD	Condition			Session			Participant		
		A	B	p	First	Second	p	Workers	Students	p
AWWL	49.9±14.5	67.4	65.8	0.239	67.4	65.8	0.237	64.9	68.7	0.295
MD	70.5±22.5	70.7	70.2	0.806	69.8	71.1	0.543	64.9	77.4	0.017*
PD	21.4±25.8	22.9	19.9	0.212	21.2	21.6	0.855	19.0	24.4	0.393
TD	57.3±28.7	59.8	54.9	0.094	56.6	58.1	0.626	60.2	53.7	0.358
OP	63.8±26.0	36.4	36.0	0.928	31.0	41.4	0.010*	37.7	34.2	0.584
EF	73.9±18.1	73.3	74.5	0.595	72.2	75.6	0.144	73.3	74.7	0.742
FR	47.9±23.1	48.1	47.6	0.870	48.0	47.8	0.942	44.7	51.9	0.200

*Notes*: * p<0.05. AWWL: Adaptive weighted workload; MD: Mental demand; PD: Physical demand; TD: Temporal demand; OP: Own performance; EF: Effort; FR: Frustration.

#### Flow state and personality

The average scores for the FSS and DJR are shown in Table 6. Data of a participant whose record was incomplete were excluded. There were over 90% of exceeded threshold for “Concentration” and approximately 80% exceeded the threshold for “Clear goals” and “Loss of self-consciousness” items (see Table 6). The mean total score was slightly above the threshold but only about 37% of the trials exceeded the threshold. Other subscale scores were lower than the threshold, and “Unambiguous feedback” and “Balanced challenge and skill” scores were very low. The total flow score was not significantly different between conditions, order, or type of occupation. The autotelic experience subscale yielded significantly higher scores for the second session (p = 0.02), and the self-oriented experience was significantly higher for working adults (p = 0.003). DJR was significantly shorter in the first session than the second (p = 0.037); overall, DJR was perceived as shorter than the actual work time. The results of the correlation matrix showed that the self-oriented experience score was significantly positively correlated with the Hard driving—Time urgency (H-T) aspect of the KG questionnaire (r = 0.339, p = 0.050, 1-β = 0.512). There was also a positive significant correlation between the concentration score and the objectivity criterion of the YG test (O: r = 0.340, p = 0.049, 1-β = 0.515). However, the correlation coefficient values and the power results do not indicate a robust relationship between the FSS and the two personality tests (S2 Table).

**Table 6:** Results of the flow scores and Duration judgment ratio.

		mean	SD	Number of thresholds exceeded (n = 68)	
FSS Total score (36–252)		145.7	26.62	33	(36.8%)
**Subscale (4–28)**	Concentration	22.2	4.11	64	(94.1%)
	Clear goals	19.6	4.57	53	(77.9%)
	Loss of self-consciousness	15.5	5.01	25	(77.9%)
	Transformation of time	15.3	4.81	32	(47.1%)
	Action-awareness merging	15.2	5.11	30	(44.1%)
	Sense of control	15.8	3.97	24	(35.3%)
	Autotelic experience	15.7	4.90	23	(33.8%)
	Unambiguous feedback	13.8	4.42	19	(27.9%)
	Challenge-skill balance	12.6	4.45	12	(17.6%)
**DJR score (0–100)**		23.8	25.3	58	(85.3%)

*Notes*: DJR: Duration Judgment Ratio; The numbers in parentheses are the minimum and maximum values for each item.

The cooperativeness criterion (Co) of the YG test had a significant correlation with task performance (number of unfinished e-mails: r = 0.382, p = 0.026, 1-β = 0.596, completed e-mails: r = 0.372, p = 0.03, 1-β = 0.621). Nevertheless, the power of these results was also small.

Significant negative correlations were found for the general activity criterion (G) of the YG test with task performance (number of unfinished e-mails: r = -0.483, p = 0.004, 1-β = 0.844, completed e-mails: r = -0.523, p = 0.002, 1-β = 0.905, characters entered: r = -0.424, p = 0.012, 1-β = 0.723). Similarly, significant correlations were noted between the number of input letters and Type A (r = 0.421, p = 0.013, 1-β = 0.716) as well as between the rhathymia criterion (R) and the number of letters per e-mail (r = -0.448, p = 0.008, 1-β = 0.839), see S2 Table.

The mean and standard deviation of the DJR score for all trials (n = 68) was presented in Table 6. The results for each session were as follows: Session 1: mean±SD = 18.8±23.5, Session 2: mean±SD = 28.8±26.4. A one-sample t-test analysis indicated that the DJR scores were significantly shorter than the 50-point threshold (indicating equal perception of real and perceived time) in both sessions.

The correlation matrix between NASA-TLX and FSS can be found in Table 7. Due to the large number of trials (n = 68), careful consideration is required when assessing the significance of the correlations. In this study, correlation coefficients exceeding ±0.39 (p<0.001) were considered significant. Regarding the items with high scores, "Concentration" was correlated with Physical demand and Effort, and "Loss of self-consciousness" was correlated with Own Performance and Adaptive WWL. Regarding of the items that were experienced by approximately half of the participants, “Transformation of Time” and “Action-awareness merging” have significant correlation with Mental demand. Conversely, there were significant negative correlations between the items related to low scores (such as “Unambiguous feedback” and “Challenge-skill balance”) and Frustration.

**Table 7:** Correlation matrix between flow score and NASA-TLX.

	MD	PD	TD	OP	EF	FR	AWWL
FSS Total score	-0.109	-0.227	-0.050	-0.443***	0.121	-0.151	-0.256*
Concentration	0.098	-0.446***	-0.014	0.001	0.413***	-0.080	0.020
Clear goals	-0.173	-0.457***	-0.072	-0.223	0.137	-0.097	-0.229
Loss of self-consciousness	-0.352**	0.101	-0.371**	-0.478***	-0.091	-0.151	-0.446***
Transformation of time	0.520***	0.028	0.491***	0.142	0.177	-0.233	0.433***
Action-awareness merging	-0.458***	0.072	-0.124	-0.528***	-0.192	-0.134	-0.439***
Sense of control	-0.124	-0.283*	-0.078	-0.295*	0.039	0.324**	-0.260
Autotelic experience	-0.109	-0.117	-0.112	-0.465***	0.191	-0.066	-0.230
Unambiguous feedback	0.043	-0.174	0.049	-0.300*	0.109	-0.450***	-0.103
Challenge-skill balance	-0.019	-0.152	-0.042	-0.360**	-0.018	-0.514***	-0.190

Notes:

* p<0.05

** p<0.01

*** p<0.001.; DJR: Duration Judgment Ratio; AWWL: Adaptive weighted workload; MD: Mental demand; PD: Physical demand; TD: Temporal demand; OP: Own Performance; EF: Effort; FR: Frustration.

## Discussion

### Field research: Identifying daily work

With regard to the type of work, the frequency of appearance was high in the following order: creation work, interpersonal work, and input data work. In particular, more than 70% of the participants performed creation and interpersonal tasks, indicating that they are common responsibilities for desk workers. Furthermore, these two tasks were more temporarily stressful to the participants than the other tasks (Fig 2) and appeared significantly higher in the group with a moderate WFun score. According to the Special Survey on Industrial Safety and Health by the Ministry of Health, Labour and Welfare [25], interpersonal relationships always rank high as a strong stress factor in the workplace, which is consistent with the results of this study. Since supporting worker mental health is among the primary goals of the present research, it was considered essential to incorporate interpersonal factors in the development of the mental workload. In the advanced WFun score group, creation and interpersonal work accounted for more than 60% of the tasks (Fig 4), suggesting that they may be related to chronic stress and fatigue. In summary, we consider that developing mental tasks based on creative content, such as in creation tasks, and incorporating a communication component could help mimic practical tasks.

### Proposing a mental workload modeled on daily work

We considered the e-mail-writing task to be suitable as a practical task that is creative and one that includes a communicative element. In this task, a control condition could be set up by having all participants compose e-mails for the same situation within a time limit. The degree and experience of task difficulty could differ depending on individual skill or experience. Thus, in our study, six pattern situations were prepared and all participants were free to choose the order in which they created the e-mails. Internet searches were not available, as it is recommended that the work be conducted offline as much as possible to ensure concentration on the task at hand [26]. Further, physiological, subjective, and analytical considerations in addition to behavior are necessary for developing a mental task for the laboratory experiment [6]. Specifically, the following two conditions are necessary: (1) physiological signal measurements should not be disturbed, and (2) task performance and subjective assessments should be recorded. Regarding the former element, the e-mail composition task can be performed using only a mouse and keyboard and does not seriously interfere with the measurements of physiological signals. When considering the use of ICT devices, it is currently difficult to continuously measure certain physiological signals such as blood pressure and plethysmography in daily life because these measures need restrained one hand or finger. However, these measurements can be covered by changing the measurement position [27, 28] or by using other methods of estimation based on autonomic nervous system activity [29, 30], so it is expected for the future development of ICT devices. The NASA-TLX and flow evaluations were recorded by mouse operation as subjective assessments of the mail-creation task. Task performance can be estimated in terms of the number of completed e-mails, input characters, and typos. To verify the effect of feedback to participants that contributes to inducing a flow state, the specification allows the display and non-display of task performance to be switched at each experimental trial. However, there was no significant difference in flow scores between Session 1 and Session 2; therefore, this validation was not successful. Effective feedback methods for workers should be further investigated. In this study, participants were informed that it was not necessary to complete e-mails for all six patterns of situations within the time limit, to reduce time pressure and allow the participants to concentrate on the task. This task was flexible and versatile in that the degree of difficulty could be adjusted according to each participant by setting the suitable content/situation for e-mail development, number of patterns, and time limit.

### Validation of the mental workload

We found no difference in the degree of difficulty between the situation conditions (A and B) or the characteristics of the participants (working adults and students); thus, the settings for the e-mail-creation task were appropriate. The average difficulty score was 3.78 (maximum 6 points), indicating that the task was difficult, but not impossible, and that the settings were able to satisfy the flow condition. In the experiment, participants rated the difficulty of each situation before the e-mail-creation task. Post-experimental reports from some participants indicated that the difficulty score was higher in the second trial regardless of the task performance because the task did not proceed as expected in the first trial. The task performance and Own performance scores of the NASA-TLX were significantly higher, suggesting that the task was executed more smoothly in the second trial.

Although the number of initiatives and completed projects were significantly higher for working adults than for students, the number of words per e-mail was higher for students. It can be inferred that working adults are familiar with writing e-mails in their daily work and have the skills to convey requirements in concise sentences. Another factor is that the content of this survey was set up with company employees in mind. For student participants, it is necessary to consider the setting of club activities or classes.

Considering a physiological measurement experiment, the participants must continue to work at all times during the measurement. In other words, they must avoid finishing all tasks before the measurement is completed. In this study, participants were free to choose not to perform the tasks that they found difficult, which is not the case in real work settings. For example, it may be practical to include a setting where a task needs to be started but can be postponed and completed later.

The NASA-TLX did not show high Frustration or AWWL, but did reveal high Mental demand and Effort, suggesting that this task has the property of inducing a flow state. Although the flow score was only moderate, the subscale scores indicated that the task was concentrated and focused, with clear goals. However, the subscales for clear feedback and balance between challenge and skill were rated low.

The correlation matrix (S2 Table) between personal characteristics and flow or work performance indicates that the flow state may be related to properties of the task and/or participants’ performance rather than personal characteristics. The relationship between flow and the NASA-TLX (Table 7) also supports this possibility. These results indicate that everyone can be in a flow state, regardless of individual characteristics. Detailed analysis of the flow-induced component showed that higher scoring FSS subscales were associated with Physical demand, Own performance, and Effort of NASA-TLX. It should be noted here that Own Performance and several subscales of FSS were negatively correlated. The Own Performance showed a negative correlation with the number of completed e-mails, number of input letters, and DJR, indicating that participants did not rate the number of e-mails completed, but rather based on the degree to which they were able to meet the goals they had set for themselves. The subscales with moderate flow scores were negatively correlated with Mental demand and Own Performance. The high mean score of Mental demand may have interfered with Action-awareness merging and loss of self-consciousness. Items with low scores (Challenge-skill Balance and Unambiguous feedback) were negatively related to Frustration, suggesting that not being able to complete the task as expected may have reduced flow induction. Overall, our proposed task is characterized by low physical activity, high Mental demand, Effort, and Concentration, and these factors contribute to flow induction. However, too much Mental demand or Frustration due to stuckness in the task may reduce the flow state. An important condition for inducing a flow state is that one’s skill and the task difficulty level must be in high proportion [12]. If the difficulty of the challenge is too high compared to one’s skill, it could cause anxious feelings or stress, and if it is too low, it could lead to boredom [31]. Furthermore, if both the levels of difficulty and skill are low, participants may fall into a state of apathy or indifference. Kuraoka et al. [32] reported cases in which participants underestimated their own abilities and competencies even when they acquired sufficient skills. The same tendency may have presented in this study, but it is also possible that the task difficulty level was too high in relation to the participants’ skills. Consequently, we believe that it is effective to adjust the level of difficulty according to the skill level of each worker and to provide support such as real-time monitoring of progress and appropriate hints. Although it is challenging to set the difficulty level of the task because of individual differences in participant skills, it is necessary to improve the task by allowing participants to choose from multiple difficulty conditions, as executed in our study, and by presenting situations according to their task performance.

In the second trial of this study, work performance was presented to the participants, but feedback was not effective. The method of presenting feedback on ongoing activities is an important topic for future research. No correlation was found between the flow state and personality traits, suggesting that the flow state may be experienced by anyone under the right conditions. We consider that the proposed mental task is useful for physiological measurement experiments to induce a flow state because 1) it is practical, 2) participants can work toward a goal in a concentrated manner, 3) the difficulty level can be adjusted according to the participants, and 4) it does not interfere with biological measurements. For a task to induce a flow state in several participants, the adjustment of the difficulty level for each individual and continuous feedback should be considered in future studies.

## Conclusion

This study aimed to propose a practical mental workload exercise, by developing an e-mail-creation task based on a field survey of desk workers. The developed task was evaluated for its usefulness by examining the relationships between subjective assessment, task performance, degree of flow state, and individual characteristics. The results showed that the developed mental task constitutes practical work that can be used for concentrated efforts toward a goal. Furthermore, the tasks had the property of inducing a flow state. The results also suggest that adjustment of the task difficulty level and effective feedback methods are necessary to induce the flow state more effectively. In the future, we plan to verify the usefulness of this mental workload task model through physiological measurement experiments for estimating mental stress and the flow state.

## Supporting information

S1 Table Situations of e-mail creation. (PDF)

S2 Table Correlation matrix between personality tests and FSS or task performance. (** p<0 .01, * p<0.05, n = 34).
(PDF)

S1 Fig Sample of task screen. (PDF)
