# Supplementary material for: Mental workload task modeled on office work: Focusing on the flow state for well-being
Source: PLoS One. 2023 Sep 6;18(9):e0290100. doi: 10.1371/journal.pone.0290100 (PMC10482285; doi:10.1371/journal.pone.0290100)
Supplement: S1 Table — (PDF) [file pone.0290100.s001.pdf]

S1 Table. Situations of e-mail creation.

A1	This morning, you have a high fever of 39°C and a headache. In the five years since you joined the company, you have never taken a sick day, and even when you were feeling a little sick, you would still come to work. However, today you are too sick to go to work, and there is a high possibility that you might spread the illness to other employees, so you made an appointment to consult a doctor at a hospital near your home. You would like to inform your supervisor, Mr. Kato, that you will be absent today, but Mr. Kato is currently in an early morning meeting and it is difficult to contact him by phone considering the time of the examination. Therefore, you decide to send an e-mail to Mr. Kato before the appointment.
A2	You are an employee of the Product Development Department and are working on a joint development project with Sangyo Gijutsu Kaisha. You have decided to file a patent application for an idea you conceived, and you have been asked to include Mr. Kubota of Sangyo Gijutsu Kaisha as an inventor. After checking with the expert, he suggested that you decide on a percentage, since you will have a future relationship with Mr. Kubota, although there is no need to include him since he was not involved in the conception of the idea. You believe that the appropriate percentage values for you and Mr. Kubota will be 90% and 10%, respectively, but the other party seems to think that 50% each is suitable. Therefore, you would like to e-mail Mr. Yoshida, the general manager of Sangyo Gijutsu Kaisha, to discuss the matter so that there are no bitter feelings.
A3	You are the project leader for the development of a product related to health care called Sun Management. The project deadline is approaching, but progress is stalled. You ask your colleagues in the same department if they could help you, but they tell you that they are too busy with other tasks to extend their support. Therefore, you decide to write an e-mail to Mr. Okimura, head of the Product Development Department, to discuss the project with him, including the need for additional staff.
A4	The accounting software in the company has been in use for more than 10 years. The accounting department has been complaining that the software is very slow and freezes frequently, making it difficult to perform accounting processes, and the company has been considering the introduction of new software. After researching accounting software on the Internet with the supervisor, Software Accounting Company was selected as a candidate because of its extensive functions and good reputation by word of mouth. Thus, you decide to send an e-mail to the department in-charge of Software Accounting Company to request information materials.
A5	You and a colleague have decided to go on a business trip for four days and three nights in a short notice. If possible, you would like to travel by SAN airlines, but the total budget must be within 150,000 yen. Although it is possible to change the airline, it is preferable to adjust the budget for the destination to stay within the limit. If the budget is inevitably exceeded, it is not impossible to shorten the length of the business trip or apply for some additional budget. You have to send an e-mail to Mr. Kono at the travel agency to request a travel expense estimate that meets this request, but there are only 10 days left before departure. In the unlikely event that you apply for an additional travel budget, you would need three days to settle the company's account.
A6	You are the organizer of a welcome party for Mr. Takekawa, a mid-career hire. Mr. Takekawa seems to like Italian food these days and is looking for a restaurant near his office that he can casually visit alone. There is one popular Italian restaurant within walking distance from the office, but it is hard to make reservations there. For the time being, you decide to use the coordination tool (https://chosei.event.co.jp) to coordinate the schedule within the company and send an e-mail to all members of the same department so that they can respond. The deadline for responses shall be July 18, and the e-mail shall be sent using the mailing list.

B1	You have been working for a new company for five days. After joining the company, you applied to the Information System Section for an e-mail address, and today, a company e-mail address (orio-u@sangyo.co.jp) was issued to you and your settings have been completed. In addition, looking at the internal contact network distributed to you, it seems that this company has a mailing list for each department. Therefore, you decide to use this mailing list to inform people in the same department of your e-mail address.
B2	Last week, Ms. Hanako Kyushu resigned from the same department. A client called us and discovered that Ms. Hanako had not informed the client about her resignation and that she had forgotten to hand over some important documents. An internal investigation revealed that no record of Ms. Hanako's work was kept, and no details about the important documents or whether they had been prepared could be determined without contacting Ms. Hanako. You receive an e-mail from the section chief, Mr. Tanaka, who is on a business trip, asking you to take over Hanako-san's duties. You could not get along with Hanako-san, so you decide to reply to the e-mail refusing the chief's request.
B3	You are an employee of a laptop computer sales company. Yesterday, a customer (Mr. Ueda) placed an order for a blue laptop computer, which you immediately shipped. It is scheduled to be delivered today, but while sorting through the slips, it was discovered that you sent a silver product by mistake. Currently, there is no blue product in stock, and the new arrival date has not yet been determined, and even if it does arrive, it will take at least two weeks at the earliest. After consulting with your supervisor, you are allowed to give a 5% and 10% discount for the blue and silver color laptops, respectively. However, if Mr. Ueda wishes to receive the blue color laptop, the silver product must be returned unopened (cash on delivery). You are to send an e-mail to Mr. Ueda apologizing for the inconvenience and discussing how to handle the situation in the future.
B4	You have come up with a development idea for a new product, and it has been recognized in a national project with brilliant results. The product you developed sold very well, and you were given the Chairman's Special Award for your contribution to the company. In addition to the special bonus, you received a week's special vacation and went to work for the first time in a while. You decide to send a thank-you e-mail to the same project members (three of them) who coordinated the work.
B5	You are currently on the train to work, but the train has stopped due to an accident and you are not sure if you will be able to make it to the morning meeting. The meeting will be attended by you, your supervisor (Mr. Sato), and several people from other departments. The meeting materials were printed and prepared on your desk yesterday, but before the meeting, you need to check the final number of participants and make copies for them. You call the office to inform your supervisor about this situation, but no one is at work yet and you cannot get through. You decide to send an email to your supervisor in a hurry.
B6	Your company is developing products related to health care, and this time you have decided to take on the challenge of incorporating big data and artificial intelligence (AI) into your products. However, since there is no employee or department familiar with such matters, you consulted with your boss and were able to form a business alliance with a specialized vendor, Sampo Co. For six months, you have been meeting several times with Mr. Taro Sangyo of Sampo Company to discuss the matter, and things are progressing smoothly. The other day, you had another meeting regarding the gender and age range of the product's target customers, so you decide to send him an e-mail to thank him for the meeting.

Note: The persons, organizations, and names appearing in this situation are fictitious and not real.
