# Supplementary material for: Mental workload task modeled on office work: Focusing on the flow state for well-being
Source: PLoS One. 2023 Sep 6;18(9):e0290100. doi: 10.1371/journal.pone.0290100 (PMC10482285; doi:10.1371/journal.pone.0290100)
Supplement: S2 Table — (** p<0 .01, * p<0.05, n = 34). (PDF) [file pone.0290100.s002.pdf]

S3 Table. Correlation Matrix between personality tests and FSS or task performance. (p<0 .01, * p<0.05, n=34)**

	FSS										Task performance			
	Total score	Clear goals	Unambiguous feedback	Self-oriented experience	Concentration	Sense of control	Transformation of time	Autotelic experience	Balanced challenge and skill	Loss of self-consciousness	Unfinished e-mails	Completed e-mails	Characters entered	Characters per e-mail
TypeA	-0.078	-0.158	0.021	0.155	-0.233	-0.108	-0.043	-0.056	-0.148	0.056	0.234	0.262	**0.421***	0.264
A-H	0.042	-0.032	0.155	0.131	-0.102	-0.082	0.148	0.014	-0.135	0.102	0.316	**0.362***	**0.395***	0.152
H-T	-0.066	-0.085	-0.125	**0.339***	-0.133	-0.003	-0.319	0.007	-0.252	0.127	0.260	0.272	**0.393***	0.191
S-P	-0.186	-0.263	-0.082	-0.049	-0.293	-0.163	-0.102	-0.145	-0.008	-0.027	-0.038	-0.050	0.190	0.269
D	0.260	0.202	0.289	0.109	-0.007	0.204	0.253	0.157	0.149	0.170	0.318	0.304	0.188	-0.086
C	-0.006	0.014	0.068	-0.166	0.008	0.013	-0.071	0.113	-0.093	0.094	0.199	0.223	**0.372***	0.329
I	-0.066	0.144	-0.090	-0.208	0.199	-0.066	-0.119	-0.029	-0.177	-0.003	0.196	0.252	0.066	-0.092
N	0.074	0.100	0.070	-0.074	0.088	0.106	0.044	0.129	0.043	-0.044	0.186	0.232	0.287	0.290
O	0.230	0.039	0.158	0.242	**0.340***	0.122	0.108	0.112	0.112	0.124	0.003	-0.023	0.049	0.037
Co	-0.024	-0.076	0.043	0.049	-0.028	-0.216	0.114	0.041	-0.014	-0.092	**0.382***	**0.372***	0.179	-0.174
Ag	-0.007	0.095	-0.065	0.121	0.061	0.064	-0.274	0.136	-0.281	0.095	0.025	0.017	0.115	0.073
G	-0.071	-0.136	-0.048	0.125	-0.298	-0.006	-0.149	-0.040	0.025	0.067	**-0.483****	**-0.523****	**-0.424***	-0.118
R	0.025	-0.326	-0.047	0.208	-0.111	-0.206	0.146	0.094	0.055	0.232	-0.014	-0.043	-0.238	**-0.448****
T	0.115	-0.026	0.139	0.146	-0.044	0.132	0.267	0.102	0.094	-0.145	0.073	0.016	0.000	-0.136
A	0.184	0.273	-0.063	0.029	0.243	0.312	-0.128	0.298	0.027	0.132	-0.184	-0.207	-0.107	0.081
S	0.109	-0.055	0.106	-0.009	-0.063	0.102	0.188	0.202	0.226	-0.057	-0.171	-0.218	-0.077	0.070

Note. FSS and task performance were averaged over all trials. The subscales of the personally tests are as follows: aggression-hostility (A-H), hard driving-time urgency (H-T), speed-power (S-P), depression (D), cyclic tendency (C), inferiority feeling (I), nervousness (N), lack of objectivity (O), lack of cooperativeness (Co), lack of agreeableness (Ag), general activity (G), rhathymia (R), thinking introversion (T), ascendance (A), and social introversion (S).
