# Supplementary material for: Mental workload task modeled on office work: Focusing on the flow state for well-being
Source: PLoS One. 2023 Sep 6;18(9):e0290100. doi: 10.1371/journal.pone.0290100 (PMC10482285; doi:10.1371/journal.pone.0290100)
Supplement: S1 Fig — (PDF) [file pone.0290100.s003.pdf]

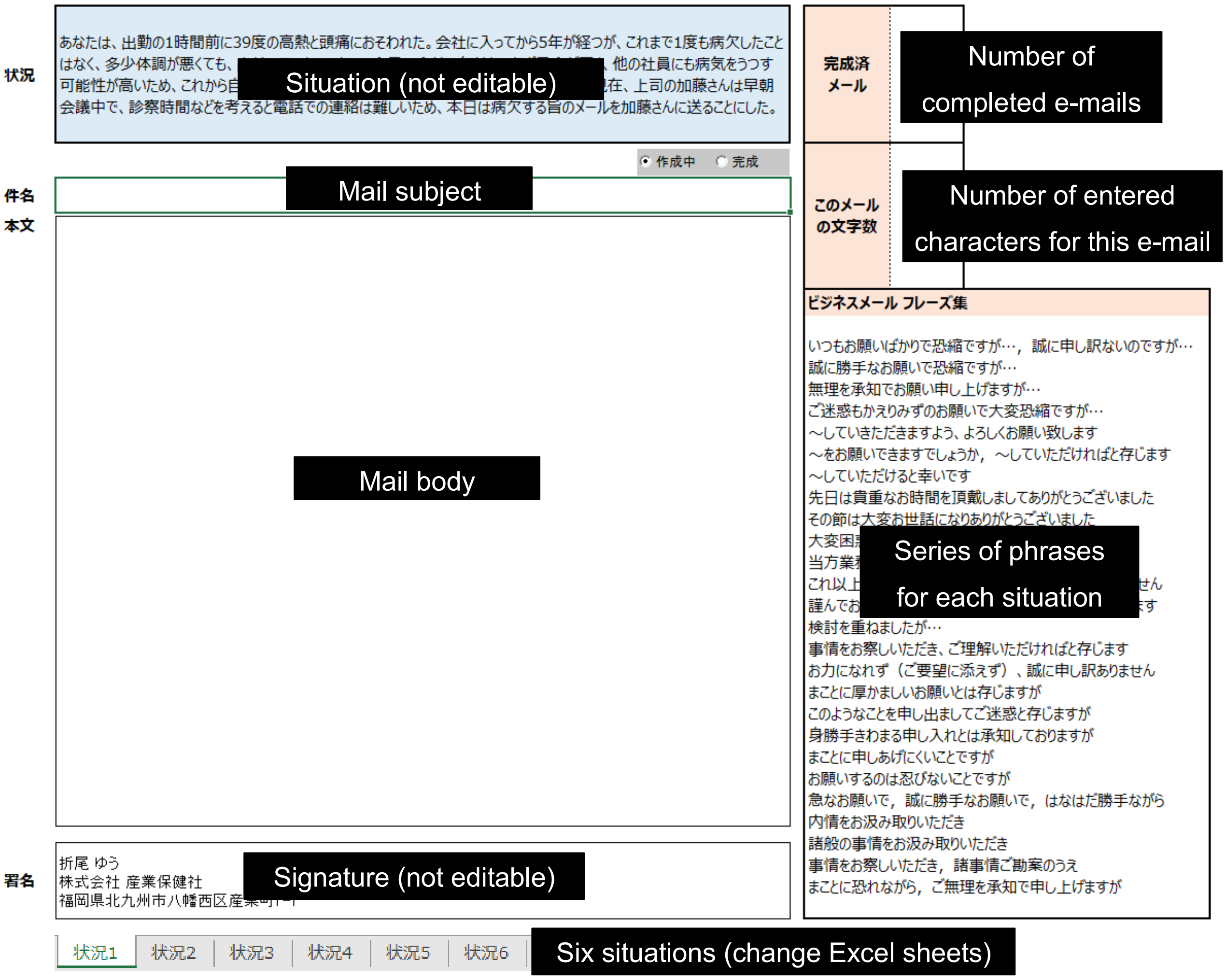

S2 Fig. Sample of task screen
